# Increased sympathovagal imbalance evaluated by heart rate variability is associated with decreased T2* MRI and left ventricular function in transfusion-dependent thalassemia patients

**DOI:** 10.1042/BSR20171266

**Published:** 2018-02-02

**Authors:** Sintip Pattanakuhar, Arintaya Phrommintikul, Adisak Tantiworawit, Sasikarn Konginn, Somdet Srichairattanakool, Siriporn C. Chattipakorn, Nipon Chattipakorn

**Affiliations:** 1 Cardiac Electrophysiology Research and Training Center, Faculty of Medicine, Chiang Mai University, Chiang Mai 50200, Thailand; 2 Department of Rehabilitation, Faculty of Medicine, Chiang Mai University, Chiang Mai 50200, Thailand; 3 Department of Medicine, Faculty of Medicine, Chiang Mai University, Chiang Mai 50200, Thailand; 4 Department of Biochemistry, Faculty of Medicine, Chiang Mai University, Chiang Mai 50200, Thailand; 5 Department of Physiology, Faculty of Medicine, Chiang Mai University, Chiang Mai 50200, Thailand; 6 Department of Oral Biology and Diagnostic Sciences, Faculty of Dentistry, Chiang Mai University, Chiang Mai 50200, Thailand; 7 Center of Excellence in Cardiac Electrophysiology Research, Chiang Mai University, Chiang Mai 50200, Thailand

**Keywords:** heart rate variability, iron overload cardiomyopathy, MRI T2*, Transfusion dependent thalassemia

## Abstract

Early detection of iron overload cardiomyopathy is an important strategy for decreasing the mortality rate of patients with transfusion-dependent thalassemia (TDT). Although cardiac magnetic resonance (CMR) T2* is effective in detecting cardiac iron deposition, it is costly and not generally available. We investigated whether heart rate variability (HRV) can be used as a screening method of iron overload cardiomyopathy in TDT patients. HRV, evaluated by 24-h Holter monitoring, non-transferrin bound iron (NTBI), serum ferritin, left ventricular (LV) ejection fraction (LVEF), and CMR-T2* were determined. Patients with a cardiac iron overload condition had a significantly higher low frequency/high frequency (LF/HF) ratio than patients without a cardiac iron overload condition. Log-serum ferritin (*r* = −0.41, *P*=0.008), serum NTBI (*r* = −0.313, *P*=0.029), and LF/HF ratio (*r* = −0.286, *P*=0.043) showed a significant correlation with CMR-T2*, however only the LF/HF ratio was significantly correlated with LVEF (*r* = −0.264, *P*=0.043). These significant correlations between HRV and CMR-T2* and LVEF in TDT confirmed the beneficial role of HRV as a potential early screening tool of cardiac iron overload in thalassemia patients, especially in a medical center in which CMR T2* is not available. A larger number of TDT patients with cardiac iron overload are needed to confirm this finding.

## Introduction

Thalassemia is a hereditary hematologic disorder resulting from abnormal production of the globin chains of hemoglobin. These changes can cause a variety of clinical pictures, from normal red blood cell concentration to stillbirth due to severe hemoglobinopathy. Transfusion-dependent thalassemia (TDT) is the most severe form of thalassemia in which regular red blood cell transfusions are a necessity for survival [1]. A definitive treatment of thalassemia is stem cell transplantation, which is limited in its availability due to the difficulty in locating a compatible donor, so regular blood transfusion is still a mainstream treatment of TDT [2]. However, long-term red blood cell transfusions cause peripheral blood iron overload, resulting in deposition and damages in iron-sensitive vital organs, including the heart [3]. Evidence shows that the leading cause of deaths in TDT patients is iron overload cardiomyopathy [4]. Although iron chelators are available, the success rate of chelation is limited because iron overload cardiomyopathy can be reversible only if the initiation of intensive chelation is early enough [5,6]. Therefore, an early detection of iron overload cardiomyopathy is an important strategy in the prevention of mortality in TDT patients.

Several methods including the measurement of both biochemical and radiological parameters have been proposed for use as screening tools for iron overload cardiomyopathy in TDT. Serum ferritin is one of the biomarkers frequently used for iron overload detection [1,7]. However, since serum ferritin is an acute-phase response protein, it has a low specificity for the iron overload condition [1,8]. Non-transferrin bound iron (NTBI) is a low molecular weight and free-form iron which can be detected in plasma only when serum transferrin is fully saturated [9]. NTBI has a high specificity to the iron overload condition, and the correlation between NTBI and vital organ damage has been recently demonstrated [10,11]. Therefore, NTBI can be one of the ideal biochemical parameters used to represent cardiac iron status. However, it has some limitations in generalized usage, specifically the high cost and complicated laboratory procedures [12]. Cardiac MRI T2* (CMR T2*) is a gold-standard diagnostic tool in the recognition of iron overload cardiomyopathy, since it has been shown to have a positive correlation with cardiac function in TDT [13–15]. However, due to its high cost and limited availability, especially in developing countries, CMR T2* might not be the best general screening tool for iron overload cardiomyopathy.

Heart rate variability (HRV), an easily performed, reliable, low cost, and non-invasive cardiac electrophysiologic test, can be used to evaluate cardiac autonomic function in patients with heart disease [16], including post-myocardial infarction [17], heart failure [18], and also iron overload cardiomyopathy in TDT patients [19,20]. Recently, we have demonstrated that depressed HRV has a positive correlation with CMR T2* in non-TDT [12] and pediatric TDT patients [21]. However, these correlations in adult TDT patients have not been elucidated yet. Moreover, those previous studies also had a limitation due to the very low prevalence of iron overload cardiomyopathy, which is defined by CMR T2* ≤20 ms. Therefore, the aim of the present study was to investigate the correlations between HRV parameters and cardiac dysfunctions, including cardiac iron overload status and ventricular contractile function, as well as to compare the diagnostic ability of HRV and biochemical markers such as serum ferritin and plasma NTBI in adult TDT patients. We hypothesized that the existence of a depressed HRV is positively correlated with cardiac iron overload status but negatively correlated with ventricular contractile function, and HRV is a better indicator of cardiac dysfunction in TDT patients than either serum ferritin or NTBI.

## Methods

### Study protocol

The study protocol was approved by the Institutional Ethics Committee of the Faculty of Medicine, Chiang Mai University, Chiang Mai, Thailand and were in accordance with the current version of the Helsinki Declaration. The inclusion criteria included: being diagnosed with TDT, having regular red blood cell transfusions at least once a month after diagnosis, being in the age range from 18 to 50 years and having no infectious diseases or acute illnesses during the investigation. The exclusion criteria included patients with a contraindication to MRI, and clinical evidence of other secondary causes of pulmonary hypertension, including HIV infection, hepatitis virus infection, collagen vascular diseases, cirrhosis, chronic obstructive airway diseases, and acquired heart disease associated with pulmonary hypertension. Patients with neurological diseases causing dysautonomia, severe depression, or chronic inflammatory diseases as well as patients, who were the known cases of the disease that can be the causes of diastolic and systolic cardiomyopathy (e.g. diabetes, hypertension, or coronary heart disease) were also excluded. Medical records of the patients were reviewed to amass epidemiological data, transfusion history, and comorbidities. The body weight, height, and body mass index (BMI) were measured in each patient. Routine laboratory tests, serum ferritin, plasma NTBI, echocardiography, 24-h Holter ECG recording for HRV analysis and CMR T2* were investigated and analyzed at the time of entry. Serum ferritin and plasma NTBI were determined using standard techniques as described previously [21,22].

### Echocardiographic studies

Standard 2D and transthoracic Doppler echocardiography was performed at rest in all enroled patients with Philips iE33 (Philips Healthcare, Bothell, WA, U.S.A.). Left ventricular (LV) average S.D. of all 5-min R–R intervals were measured, and LV ejection fraction (LVEF), stroke volume, cardiac output, and fractional shortening (FS) were determined based on the calculation of LV volumes by the method of discs, following the recommendations by the American Society of Echocardiography (ASE), and using apical two- and four-chamber views to measure cardiac function [23,24]. Impaired systolic LV dysfunction (LVEF <55% or LVFS <35%) was also determined in TDT patients [24]. Diastolic function was evaluated from mitral inflow velocities (E-wave, A-wave, E/A ratio) and deceleration time (DT) using pulsed wave (PW) Doppler in the apical four-chamber view. PW tissue-Doppler velocities were acquired at end-expiration, in the apical four-chamber view, with the sample positioned at the lateral mitral annulus, measuring early diastolic (E′) and late diastolic (A′) velocities and calculating the E/E′ ratio. Diastolic function was investigated using E/A ratio, DT, and E/E′ according to the current guidelines [25].

### HRV measurement

HRV was evaluated using the SEER Light Holter system (GE Healthcare, Milwaukee, WI, U.S.A.). A lead II ECG was continuously recorded for 24 h. Prior to the HRV measurement, either exercise or caffeine consumption was prohibited in all the participants. The computer software MARS version 7, GE Healthcare, Milwaukee, WI, U.S.A., was used to scan for rhythm disturbance and to detect and label each QRS complex. Excessive noise and artifacts were noted, and ectopy was quantitated. The time-domain analyses including average heart rate, average R–R intervals (NN), standard deviation (S.D.) of the R–R intervals over a 24-h period (SDNN), S.D. of all 5-min mean R–R intervals (SDANN), average S.D. of all 5-min R–R intervals (ASDNN), the percentage of R–R intervals with more than 50-ms variation (pNN50), and the square root of mean squared differences of successive R–R intervals (rMSSD). The frequency domain analyses were determined with the same analytical software using Fast-Fourier transform analysis. The obtained frequency domain indices were the total power (0–0.4 Hz), high-frequency power (HF, 0.15–0.4 Hz) spectral density, low-frequency power (LF, 0.04–0.15 Hz) spectral density, and very LF (0.003–0.04 Hz) spectral density. Total power expresses the magnitude of the entire HRV, whereas HF power reflects the parasympathetic tone, and LF power indicates the sympathovagal interactions [16]. These power spectral densities were described in absolute units (ms^2^). The designated physician, who operated and fitted the Holter monitor to the patients, was blinded to the patient information. The subjects were fitted with the Holter monitor after blood sample collection at the Holter unit of the Faculty of Medicine, Chiang Mai University. In the present study, none of the patients included in the study took medication such as β-blockers, calcium channel blockers, statins, and ACE inhibitors, which could affect the HRV [26].

### MRI

Magnetic resonance acquisition was performed using a 1.5-T MR scanner (GE, HDxt, Milwaukee, WI, U.S.A.), with an eight-channel body array. For the measurements of myocardial iron overload, we used a T2 gradient-echo multi-echo sequence (25-degree flip angle, matrix: 192 × 128 pixels, 38 × 28.5 cm field of view,125-kHz bandwidth, 10.0-mm slice thickness, 1 number of excitations, 4 views per segment, 18.5-ms repetition time). A single mid ventricular short-axis view of the left ventricle was acquired at eight echo times (TEs) (1.7 ms, which increased in 2.0–2.1 ms increments) in a single end-expiratory breath-hold. CMR T2* analysis was performed on a workstation (Advantage Window 4.6, GE Healthcare) using commercial analysis software (StarMap 4.0, GE Healthcare). According to the previous study, the 95% confidence interval of a normal CMR T2* value (>20 ms) was assessed [13,27]. The values 14–20 ms were regarded as mild iron overload, values between 10 and 14 ms were regarded as moderate iron overload, and a T2* less than 10 ms was regarded as severe iron overload [13].

### Statistical analysis

Categorical variables were described using percentages of its frequency. Normally distributed numerical variables were presented using arithmetic means and S.D. Non normally distributed variables were modified by taking a natural logarithm, and then presented using geometric mean and S.D. Differences of parameters between patients with and without cardiac iron overload were compared using the independent Student’s *t*test. The correlations amongst HRV, biochemical markers, echocardiographic parameters, and CMR T2* were determined using the Pearson’s correlation coefficient. The multivariate linear and binary logistic regression analysis, forward likelihood method, as well as the receiver operative characteristic (ROC) curve were applied to determine independent predictive factors of CMR T2* values. Statistical analyses were performed using SPSS version 13.0 for Windows (SPSS Inc., Chicago, IL, U.S.A.). A *P*-value of less than 0.05 was considered statistically significant.

## Results

Sixty-six TDT patients were enroled in the present study. However, since 7 patients were not evaluated by CMR T2*, only 59 TDT patients were included in a statistical analysis. Nineteen patients (32.2%) were diagnosed with homozygous β-thalassemia. Thirty-eight patients (64.4%) were diagnosed with β-thalassemia/Hemoglobin E. One patient was diagnosed with AE Bart’s disease and the other patient was diagnosed with AE Bart’s Constant Spring (CS) disease. Clinical and biochemical characteristics, echocardiographic and HRV parameters, as well as CMR T2* values of the patients are summarized in Table 1. All continuous variables, except serum ferritin level, were normally distributed. After taking a natural logarithm (ln), the serum ferritin variable was also normally distributed. Therefore, the parametric statistical analysis was applied in all tests. The mean of number of red blood cell transfusions per year in the last 5 years in these subjects was 19.44 times. The number of patients with positive cardiac iron overload, indicated by CMR T2* ≤20 ms, were five, reflecting that the prevalence of cardiac iron overload in the present study was 8.47%. Moreover, when the subjects were divided into two groups, based on the present of cardiac iron overload, the positive cardiac iron overload group had a significantly higher ratio between LF and HF (LF/HF ratio), which indicates a sympathovagal imbalance of the heart, and higher ln-serum ferritin than the negative cardiac iron overload group (1.77 compared with 1.46, *P*=0.028 and 8.01 compared with 7.26, *P*=0.010, respectively). On the other hand, there was no difference in clinical characteristics (including age, sex, BMI, and number of red blood cell transfusions per year in the last 5 years), Hb, plasma NTBI, LVEF, or the other HRV parameters between these two groups.

**Table 1:** Demographic, clinical, biochemical characteristics, cardiac imaging parameters, time-domain and frequency-domain HRV parameters of all transfusion dependent thalassemia TDT patients (*n*=59) and comparisons between CMR T2* ≤20 and >20 groups

Parameters	All patients (*n*=59) mean (S.D.)	CMR T2* ≤20 (*n*=5) mean (S.D.)	CMR T2* >20 (*n*=54) mean (S.D.)	*P*-value
***Demographic data***				
**Age (years)**	28 (8)	23 (5)	28 (8)	0.178
**Sex (male/female) (%)**	39/61	20/80	42/58	0.389*
**Number of transfusions in last 12 months (times)**	19 (6)	18 (5)	20 (6)	0.500
***Biochemical parameters***				
**Hb (g/dl)**	7.18 (1.0)	7.3 (1.7)	7.1 (1.0)	0.645
**Hct (%)**	23.62 (3.77)	24 (5)	23 (3)	0.892
**Geometric mean of serum ferritin (95% CI) (ng/dL)**	1448 (1189, 1764)	3920 (1520, 10011)	1443 (1188, 1754)	0.011^†^
**Plasma NTBI (µM)**	6.7 (1.9)	7.41 (2.03)	6.69 (1.92)	0.435
***Cardiac imaging parameters***				
**LVEF (%)**	68 (4.2)	68 (6)	69 (4)	0.594
**LV diastolic volume (ml)**	87 (24)	77 (26)	87 (23)	0.318
**E/A ratio**	1.6 (0.6)	1.8 (0.4)	1.6 (0.6)	0.594
**TRV max**	249 (55)	252 (15)	248 (54)	0.377
CMR T2* (ms)	38 (12)	7 (3)	41 (9)	0.000^†^
Positive for cardiac iron overload (CMR T2* ≤20 ms) (%)	8.47			
***HRV-frequency domain***				
**LF (ms^2^)**	12 (5)	11 (4)	12 (8)	0.784
**HF (ms^2^)**	8 (4)	7 (5)	8 (4)	0.498
**LF/HF ratio**	1.5 (0.3)	1.8 (0.3)	1.5 (0.3)	0.028^†^
***HRV-time domain***				
**SDNN (ms)**	97 (28)	95 (26)	97 (28)	0.831
**SDANN (ms)**	89 (28)	86 (24)	90 (28)	0.763
**ASDNN (ms)**	36 (11)	36 (14)	36 (11)	0.954
**rMSSD (ms)**	20 (9)	18 (10)	21 (10)	0.256

† Significant at *P*<0.05.

All statistical analyses were performed by independent *t* test except * by Fisher’s exact test.

Abbreviations: Hb, hemoglobin; Hct, hematocrit; TRV max, maximum velocity of tricuspid regurgitant flow.

The correlation analyses between biochemical markers, echocardiographic data, HRV parameters, and CMR T2* values were determined. None of the clinical characteristics showed a correlation with biochemical markers, HRV parameters, or CMR T2*. None of the HRV parameters showed a significant correlation with log-serum ferritin or plasma NTBI. As regarding the correlation between HRV parameters and CMR T2*, only the LF/HF ratio had a significant negative correlation with CMR T2* values (*r* = −0.286, *P*=0.021). Log-serum ferritin and plasma NTBI also had a significant negative correlation with CMR T2* values (*r* = −0.341, *P*=0.008 and *r* = −0.313, *P*=0.029, respectively). However, only the LF/HF ratio parameter of HRV *did have* a significant negative correlation with LVEF.

**Figure 1:**
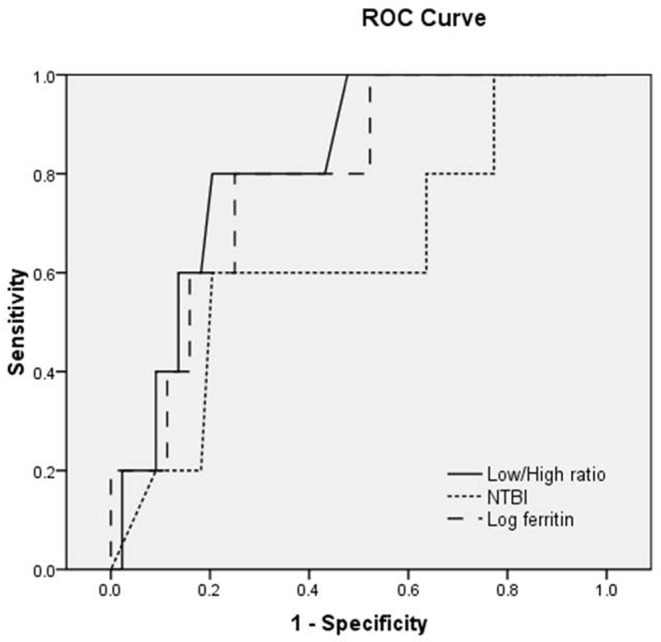
ROC curve of LF/HF ratio parameter of HRV, NTBI and log-serum ferritin for the prediction of cardiac iron deposition determined by CMR T2*

To determine independent predictive factors of the cardiac iron overload status; a multivariate logistic regression analysis, forward likelihood method, was applied. After adjustment by log-serum ferritin and plasma NTBI, LF/HF ratio parameter of HRV was the only independent predictive factor of the cardiac iron overload status (*P*=0.033, odd ratio =28.43; 95% CI =1.30–621.02). Furthermore, the area under ROC curve was used to demonstrate the predictive accuracy of the parameters. The predicted area under the ROC curve of the LF/HF ratio was in the good level (0.82 ± 0.79; 95% CI =0.67–0.98) while the predicted area under the ROC curve of log-serum ferritin and plasma NTBI were in the fair (0.79 ± 0.9; 95% CI =0.61–0.97) and the poor levels (0.63 ± 0.14; 95% CI =0.37–0.90), respectively. In addition, LF/HF ratio more than 1.49 was the best predictor for CMR T2* ≤20 ms, with 83% sensitivity and 50% specificity and its positive and negative predictive values were 16 and 96%, respectively (Figure 1).

## Discussion

The major findings of the present study were that depressed HRV, indicated by increased LF/HF ratio, showed a significant correlation with the presence of cardiac iron overload status, indicated by CMR T2* ≤20 ms. For example, the LF/HF ratio was the only predictive factor determining the present of cardiac iron overload, with the high odds ratio of 28.43. The ROC analysis also demonstrated a good accuracy of LF/HF ratio enabling a prediction of cardiac iron overload status. The LF/HF ratio also had a significant negative correlation with LVEF. On the other hand, biochemical markers, specifically serum ferritin and plasma NTBI levels, had a significant correlation with only cardiac iron overload status. These findings confirmed our hypotheses that depressed HRV is positively correlated with cardiac iron overload status but negatively correlated with ventricular contractile function, and HRV is better than both serum ferritin and NTBI in a giving clear detection of cardiac dysfunction in TDT patients. Previous studies investigated the associations between the HRV and other cardiac parameters in either adult non-TDT (NTDT) cases [12] or pediatric TDT cases [21]. However, this association has never been investigated in adult TDT patients. Our study is the first study demonstrating the correlations between HRV parameters and CMR T2*, as well as LVEF in adult TDT patients. Although most patients with positive cardiac iron overload were female (four out of five, 80%), there was no statistical significance of sexes between positive and negative cardiac iron overload groups. In addition, the multivariate regression analysis also demonstrated an independent significance of the LF/HF ratio and CMR-T2* when adjusted by sex. Therefore, sex might not contribute to the difference between positive and negative cardiac iron overload groups and this correlation could be applicable to both sexes.

The prevalence of cardiac iron overload in the present study was 8.47%, which was lower than that reported in a recent study. Aydinok et al. [28] reported in their multicenter study that the prevalence of cardiac iron overload, indicated by CMR T2* ≤20 ms, was 36.7%. Furthermore, there was absence of cardiac contractile dysfunction in this cross-sectional study. None of these subjects had an LVEF less than 45%. This finding was compatible with the results from the study of Aydinok et al. [28], which also demonstrated the absence of cardiac contractile dysfunction in their study. The decrease in cardiac iron overload prevalence compared with the studies in last 5 years, as well as the absence of cardiac contractile dysfunction in TDT patients, may reflect an effective strategy of early iron chelator administration in order to prevent the cardiac iron overload status in TDT patients [21,29].

In the present study, only the LF/HF ratio, which is a frequency-domain parameter of HRV, was negatively correlated with cardiac iron overload status. This finding emphasized the important findings from our previous studies. In non-TDT patients, we have previously demonstrated that there was a significant negative correlation between the LF/HF parameters of HRV (*r* = –0.227 and *P*=0.026), with an odds ratio of 8.71 [12]. On the other hand, our previous study investigated HRV parameters in 101 pediatric TDT patients demonstrated that only SDANN and ASDNN from time-domain parameters and LF and HF, but not the LF/HF ratio, from frequency-domain parameters of HRV showed a correlation with CMR T2* [21]. It is also notable that the correlations between HRV parameters and CMR T2* were weaker than in the present study. This inconsistent finding may be due to the difference in age of the subjects participating in each study. The median age of the subjects in the study of Silvilairat et al. [21] was 15 years while it was 27 years in the present study. Many previous studies demonstrated that HRV parameters including LF/HF ratio were age dependent [30–32]. LF/HF ratio is continuously increased from birth until 6^th^ year, corresponding with a development of sympathetic nervous system [30,31]. After 6^th^ year, LF/HF ratio gradually decreased over time, reflecting an increase in parasympathetic activity [33]. This age-dependent character may be responsible for the inconsistent finding. In addition, this difference may also be responsible for the difference in prevalence of the cardiac iron overload condition because the older the patient gets, the longer he/she lives with anemia. Combining the results from our three correlation studies, we propose that the LF/HF ratio could be the most sensitive HRV parameter for accurate prediction of the cardiac iron overload status in thalassemia patients who are in adult age.

The mechanisms involving in the depression of HRV in TDT patients could mainly be exacerbated by oxidative stress. In thalassemia patients, systemic iron overload occurred via two mechanisms, from excessive intestinal iron absorption and from transfusional siderosis [34]. Excessive iron acquisition causes saturation of transferrin-binding capacity, resulting in the presence of plasma NTBI [3]. NTBI can deposit in various tissues, such as the reticulo-endothelial system of the spleen, liver, bone marrow and in advanced cases, in the cardiac myocytes [9,35,36]. Deposition of NTBI in those tissues causes oxidative stress by enhancing reactive oxygen species (ROS) via the Haber–Weiss and Fenton reaction, leading to cellular injury from lipid peroxidation, damage to cellular proteins as well as nucleic acids, mitochondrial dysfunction, and apoptosis [15,37–39]. Previous studies have shown that the oxidative stress from many pathological conditions, including iron overload, can promote sympathovagal disturbance and cause significantly depressed HRV [40–47]. Therefore, the depressed HRV observed in the present study could be mainly caused by oxidative stress mechanisms.

**Figure 2:**
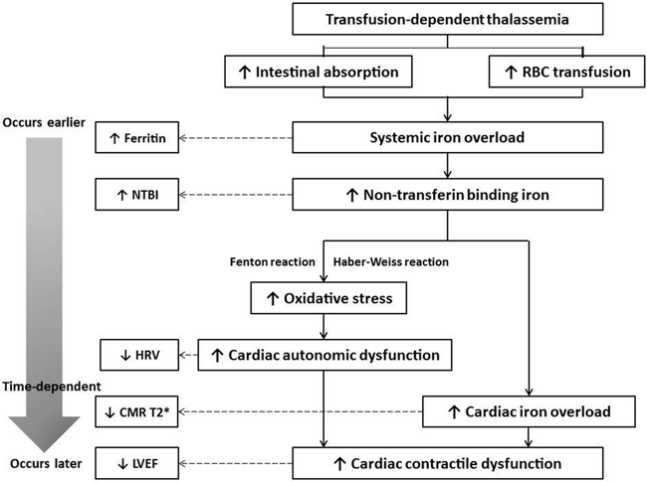
The mechanism of depressed HRV, increased serum ferritin, increased plasma NTBI, increased cardiac iron deposition, and decreased cardiac contractile function in TDT patients In TDT patients, there is an increase in intestinal iron absorption, as well as an increase in red blood cell (RBC) transfusion rate. These changes induce conditions of systemic iron overload, causing an increase in serum ferritin level. Excessive serum iron causes saturation of transferrin, an iron-binding protein, resulting in increased NTBI levels. An increase in NTBI level causes increased ROS, via the Haber–Weiss and Fenton reactions, resulting in increased oxidative stress. Oxidative stress enhances autonomic dysfunction of the heart, resulting in depressed HRV. Meanwhile, an increase in NTBI level enhances cardiac iron deposition, which is represented by decreased CMR T2* values. Eventually, chronic cardiac autonomic dysfunction and abnormal cardiac iron overload impairs cardiac contractile function, resulting in a decrease in the LVEF. The changes in these parameters are time dependent. Serum ferritin and NTBI levels are systemic parameters which occur very early in the condition and their correlation with LVEF, which is a late-stage change, is not significant. On the other hand, HRV, which is a direct change of the heart itself, occurs soon after the increase in oxidative stress, thus is correlated with both CMR T2* and LVEF. Therefore, HRV is possibly a potential screening tool for early detection of conditions of cardiac iron overload in TDT patients.

Interestingly, this is the first study demonstrating the correlations between HRV parameters and CMR T2*, as well as LVEF in adult TDT patients. Only the LF/HF ratio parameter of HRV, but not CMR T2*, was positively correlated with LVEF in the present study. This finding was different from the results of the study of Anderson et al. (2001) [13], which demonstrated a significant correlation between CMR T2* and LVEF. The difference in severity of cardiac iron overload may be responsible for these inconsistent findings. The prevalence of cardiac iron overload in the study by Anderson et al. [13] was ~70%, while it was only 8.47% in the present study. In other cardiac disorders, it was demonstrated that cardiac autonomic dysfunction was more sensitive to oxidative stress than contractile dysfunction [48–51]. Many previous studies demonstrated LF/HF ratio of HRV as the best prognostic factor of mortality in various condition, such as post-myocardial infarction [52], chronic heart failure [53], as well as severe cardiomyopathy [54]. However, only one study did correlated LF/HF with LVEF, and the result did not show a significant correlation (*r* =0.3, *P*=0.7) [55]. Since the subjects of these studies had a reduced LVEF at the time HRV was evaluated, the difference in pathophysiology of developing heart failure between intrinsic cardiac diseases (myocardial infarction, cardiomyopathy) and iron overload cardiomyopathy may be responsible for this inconsistence. This evidence addressed that the correlation between LF/HF ratio of HRV and LVEF is conceal if the patients have already a reduced LVEF at the measurement time. Therefore, our hypothesis is that in TDT patients, cardiac autonomic dysfunction occurs earlier, in mild degree of cardiac iron overloads, while cardiac contractile dysfunction occurs later, in more severe cases of cardiac iron overload. To test this hypothesis, a prospective longitudinal cohort clinical study is needed. Moreover, because the prevalence of the condition of cardiac iron overload in current clinical practice is decreasing, HRV might be a potential early screening tool of cardiac iron overload in thalassemia patients, especially in a medical center in which CMR T2* is not available. The proposed mechanism of depressed HRV in TDT, and its correlation with cardiac iron overload status as well as cardiac function is summarized in Figure 2.

## Study limitation

There were several limitations in the present study. First, there was a small sample size in the present study. Second, there was no healthy control group or an additional group with a disease associated with cardiac iron overload from another cause, such as hereditary hemochromatosis. However, because this type of disease is extremely rare in our population, the addition of this group is not possible for the present study. Next, since there were only 5 out of 59 patients who had cardiac iron overload (CMR-T2* ≤20 ms), the correlation found between HRV and cardiac iron overload had the weakest statistical power. Future studies with larger number of TDT patients with cardiac iron overload are needed to confirm this finding. Further prospective longitudinal cohort studies, with larger sample sizes, from more centers, which may include patients with other causes of iron overload and more patients with positive cardiac iron overload, are needed to increase the confidence in the results.

## Conclusion

The significant correlations between LF/HF ratio of HRV and CMR-T2* and LVEF in TDT patients indicate that HRV might have the beneficial role as a potential predictor of cardiac iron overload as well as LV contractile function in this group of patients. However, due to weak correlation and small studied population in the present study, larger population of TDT patients with positive cardiac iron overload is needed to confirm this finding.

## Abbreviations

ACE: angiotensin-converting enzyme
ASDNN: average S.D. of all 5-min R–R intervals
CI: confidence interval
CMR: cardiac magnetic resonance
DT: deceleration time
ECG: electrocardiography
FS: fractional shortening
HF: high-frequency power
HRV: heart rate variability
LF: low-frequency power
LF/HF: low frequency/high frequency
LV: left ventricular
LVED: LV end diastolic dimension
LVEF: LV ejection fraction
LVESD: LV end systolic dimension
NTBI: non-transferrin bound iron
pNN50: the percentage of R–R intervals with more than 50-ms variation
PW: pulsed wave
rMSSD: the square root of mean squared differences of successive R–R intervals
ROC: receiver operative characteristic
ROS: reactive oxygen species
SDNN: S.D. of the R–R intervals over a 24-h period
SDANN: S.D. of all 5-min mean R–R interval
TDT: transfusion-dependent thalassemia
